# Vonoprazan and high‐dose amoxicillin dual therapy for *Helicobacter pylori* first‐line eradication: A single‐arm, interventional study

**DOI:** 10.1002/jgh3.12852

**Published:** 2022-12-08

**Authors:** Soichiro Sue, Masaaki Kondo, Takeshi Sato, Hiroyuki Oka, Katsuyuki Sanga, Tsuyoshi Ogashiwa, Mao Matsubayashi, Hiroaki Kaneko, Kuniyasu Irie, Shin Maeda

**Affiliations:** ^1^ Department of Gastroenterology Yokohama City University Graduate School of Medicine Yokohama Japan; ^2^ Department of Gastroenterology Yokohama Minami Kyousai Hospital Yokohama Japan

**Keywords:** amoxicillin, dual therapy, *Helicobacter pylori*, vonoprazan

## Abstract

**Background:**

To date, no interventional trial has assessed the efficacy and safety of vonoprazan and high‐dose (500 mg four times daily, 2000 mg/day) amoxicillin dual therapy in terms of *Helicobacter pylori* eradication. We explored whether this was an appropriate first‐line treatment.

**Methods:**

This prospective, dual‐center, single‐arm interventional study was performed in Japan. Twenty *H. pylori*‐positive patients lacking any eradication history were treated with vonoprazan 20 mg twice daily and amoxicillin 500 mg four times daily (qid) for 7 days. Eradication was evaluated using a stool *H. pylori* antigen test. We evaluated safety using patient questionnaires. This study was registered in the jRCT database (jRCT031200128).

**Results:**

The intention‐to‐treat and per‐protocol eradication rates were 90% (95% confidence interval [CI] 68.3–98.8%, *n* = 20) and 94.4% (95% CI 72.7–99.9%, *n* = 18) respectively. No significant adverse event was recorded.

**Conclusion:**

Vonoprazan/high‐dose amoxicillin dual therapy can be a safe standard first‐line therapy. We are now undergoing a randomized controlled trial comparing dual therapy and vonoprazan‐based triple therapy.

## Graphical abstract

This interventional study showed the efficacy of Vonoprazan/high‐dose (500 mg four times daily) amoxicillin dual therapy. No significant adverse event was recorded.

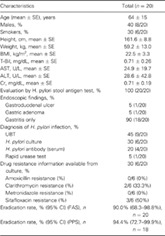

## Introduction

*Helicobacter pylori* (*H. pylori*) infections induce gastric cancer via several mechanisms,^1^ and eradication reduces gastric cancer incidence.^2^

Vonoprazan (VPZ) is the first clinically available potassium competitive acid blocker and can be the more potent gastric acid inhibition in comparison with PPIs. Recent most popular standard regimen for eradication of *H. pylori* in our country is the triple regimen with VPZ 20 mg bid, amoxicillin (AMPC) 750 mg bid, and clarithromycin (CAM) 200 or 400 mg bid for 7 days (VAC regimen). We also showed the VAC‐regimen was superior to 7‐day proton pump inhibitor (PPI)‐based triple therapy (with AMPC and CAM) in terms of eradicating CAM‐resistant *H. pylori*.^3^ In a prospective study, the eradication rate of CAM‐resistant *H. pylori* afforded by the VAC‐regimen was 82.9% (95% confidence interval [CI] 67.9–92.8%),^4^ suggesting that a VPZ‐based regimen (VPZ and AMPC dual therapy) might be useful. Furuta *et al*. retrospectively compared VPZ and AMPC dual therapy (VPZ 20 mg bid and AMPC 500 mg tid, 7 days) and a first‐line VAC‐regimen; the eradication rates were 92.9% (95% CI 82.7–98.0%) and 91.9% (95% CI 80.4–97.0%) (*p* = 0.728), respectively and the eradication rate by the dual therapy was not inferior to that of the triple therapy.^5^ Suzuki *et al*. conducted a multicenter randomized trial comparing VPZ and low‐dose AMPC dual therapy (“VA‐dual”); (VPZ 20 mg bid and AMPC 750 mg bid, 7 days) to a first‐line VAC therapy and VA‐dual was non‐inferior to VAC‐triple in the per‐protocol (PP) analysis, but not in intention‐to‐treat (ITT) analysis.^6^ This result suggests that the dual therapy is effective, but needs modification to increase the eradication rate.

As a result of considering a therapy to improve the eradication rate by modifying the dual therapy, we thought that the modification of the AMPC dose from 750 mg bid to 500 mg qid (4 times daily) is reasonable because the effects of AMPC depend on the percentage time for which the minimum inhibitory concentration is exceeded (%Time > MIC)^7^ and the plasma half‐life of AMPC is short.^8^ Thus, we performed a single‐arm, exploratory interventional study on VPZ 20 mg bid with AMPC 500 mg qid dual therapy in terms of first‐line *H. pylori* eradication. We explored whether it was appropriate to proceed to an RCT comparing the new dual therapy and the VAC‐regimen.

## Methods

### Study design

This was an open‐label, dual‐center, single‐arm interventional study of high‐dose AMPC dual therapy (with VPZ). After this assessment, we plan an RCT comparing this VPZ dose to high‐dose AMPC dual therapy and 7‐day triple therapy with VPZ, AMPC, and CAM. This study was conducted in accordance with the Clinical Trials Act of Japan and the Declaration of Helsinki. The study was conducted by the Yokohama City University Hospital and Yokohama Minami Kyousai Hospital. The study was reviewed and approved by the Yokohama City University Certified Institutional Review Board and was registered with the Japan Registry of Clinical Trials (jRCTs) (as mandated by the Clinical Trials Act of Japan) as jRCTs031200128 (https://jrct.niph.go.jp/latest‐detail/jRCTs031200128) in September 2020.

### Study population

We included male and female adults (aged >20 years) with *H. pylori* infections lacking any history of *H. pylori* eradication. In this study, we defined positive patients for a urea breath test, stool *H. pylori* antigen test, *H. pylori* culture, and anti‐*H. pylori* antibody test (blood), rapid urease test (biopsy sample), or histological diagnosis (biopsy sample) as *H. pylori* infection. This definition is in accordance with the guidelines for *H. pylori* in Japan, and clinically these diagnostic methods are generally used in Japan.^9^ Latex agglutination immunoturbidimetry kit (Denka) was used with 10 U/ml cut off for anti‐*H. pylori* antibody test, which was reported with sufficient sensitivity and specificity for diagnosis. The anti‐*H. pylori* test was performed by an external agency. Endoscopy was performed within 1 year in all patients. Patients who suspected *H. pylori* infection by endoscopy, including gastritis, was conducted the above‐mentioned *H. pylori* infection diagnosis according to national insurance‐covered medicine regulation procedure.

The exclusion criteria were an allergy to VPZ or AMPC; lactation or pregnancy; infectious mononucleosis; the use of atazanavir, rilpivirine, pimozide, ergotamine, suvorexant, lomitapide mesylate, tadalafil, ticagrelor, ibrutinib, asunaprevir, ivavradine, or venetoclax; the use of colchicine to treat liver or renal dysfunction; severe dysfunction of the liver, kidneys, or heart; and disqualification by study physicians. Written informed consent was obtained from all patients.

### Intervention

Patients received high‐dose AMPC dual therapy with VPZ: VPZ 20 mg twice daily (bid) and AMPC 500 mg four times daily (qid) for 7 days. The use of other antibiotics or PPIs was prohibited. The primary eradication rate was the (objective) outcome; the study was open‐labeled.

### Susceptibility of H. pylori to antimicrobial agents

*H. pylori* susceptibilities were assessed by culture and agar dilution of endoscopic gastric biopsy samples as described previously.^4^ The agar dilution method was used to determine the minimum inhibitory concentrations (MICs) of AMPC, CAM, Metronidazole (MNZ), and STFX. The AMPC‐resistant breakpoint was defined as ≥0.5 mg/L, the CAM‐resistant breakpoint as ≥1.0 mg/L, the MNZ‐resistant breakpoint as ≥8 mg/L, and the STFX‐resistant breakpoint as ≥0.12 mg/L, based on previous reports.^10^, ^11^, ^12^

### Outcome

We checked the efficacy and safety of the regimen to assess whether to continue with an RCT. The primary endpoint was the first‐line *H. pylori* eradication rate determined using the Full Analysis Set (FAS); stool antigen tests were performed at least 8 weeks after the end of the therapy. The FAS was defined as all patients who commenced eradication therapy except those who did not meet the study criteria. Patients lost to follow‐up or who did not undergo *H. pylori* stool antigen tests were regarded as treatment failures. The secondary endpoints were the eradication success rate determined using the per‐protocol set (PPS), and safety. All patients were told to stop VPZ, PPIs, and histamine‐2 blockers for at least 2 weeks before the *H. pylori* stool antigen test. *H. pylori* stool antigen test was performed by the hospital's internal inspection units as components of regular medical care.

### Safety

Safety was physician‐evaluated using the Common Terminology Criteria for Adverse Event ver. 5.0 (CTCAE). Also, patients completed adverse effect questionnaires (AEQs) that we have used previously.^4^, ^11^, ^13^, ^14^, ^15^ The AEQ explores fatigue, vomiting, eructation, abdominal fullness, headache, urticaria, heartburn, abdominal pain, anorexia, nausea, dysgeusia, and diarrhea; scores of 3 were strongly positive, 2 moderately positive, 1 weakly positive, and 0 negatives. All AEQs were completed before clinical examinations; reporting bias is not in play.

### Sample size calculation

We chose a sample size of 20. If the upper 95% CI was <80%, we would not proceed to an RCT. This corresponds to fewer than 12 successes. The 80% was set based on unacceptable eradication and a clinically meaningful 10% decrease from 90%, which is the eradication rate of the standard regimen.

### Statistical analysis

We calculated eradication rates with 95% CIs. We assessed whether the upper 95% CI was <80%. We calculated CTCAE and AEQ scores. All statistical analyses employed SPSS ver. 28. All authors reviewed the data and approved the final manuscript.

## Results

### Recruitment and follow‐up

We registered the study in September 2020 and enrolled patients from November 2020. The last follow‐up date was September 2021. Twenty patients with *H. pylori* infections without any eradication history received high‐dose AMPC dual therapy with VPZ. Two patients were excluded from PP analysis. One was lost to follow‐up after the completion of eradication therapy. The other was excluded because of a delayed stool antigen test (which indicated successful eradication).

### Baseline characteristics

The baseline characteristics of all patients are summarized in Table 1. All patients were endoscopically diagnosed as at least gastritis (gastroduodenal ulcer and gastritis or gastric adenoma and gastritis case included) prior to diagnostic testing with UBT, *H. pylori* culture, and anti‐*H. pylori* antibody (serum), or rapid urease test. All patients did not have comorbidities generally treated with antibiotics or antibiotic use at the recruitment. In six cases (30% of the total), AMPC‐, CAM‐, metronidazole (MNZ)‐, and STFX‐resistance data were collected prior to eradication. The AMPC resistance rate was 0% (0/6), the CAM rate was 33.3% (2/6), the MNZ rate was 0% (0/6), and the STFX rate was 50% (3/6).

**TABLE 1:** Patient characteristics and *Helicobacter pylori* eradication rates

Characteristics	Total (*n* = 20)
Age (mean ± SE), years	64 ± 15
Males, %	40 (8/20)
Smokers, %	30 (6/20)
Height, cm, mean ± SE	161.6 ± 8.8
Weight, kg, mean ± SE	59.2 ± 13.0
BMI, kg/m^2^, mean ± SE	22.5 ± 3.3
T‐Bil, mg/dL, mean ± SE	0.71 ± 0.26
AST, U/L, mean ± SE	24.9 ± 19.7
ALT, U/L, mean ± SE	28.6 ± 42.8
Cr, mg/dL, mean ± SE	0.71 ± 0.19
Evaluation by H. pylori stool antigen test, %	100 (20/20)
Endoscopic findings, %	
Gastroduodenal ulcer	5 (1/20)
Gastric adenoma	5 (1/20)
Gastritis only	90 (18/20)
Diagnosis of *H. pylori* infection, %	
UBT	45 (9/20)
*H. pylori* culture	30 (6/20)
*H. pylori* antibody (serum)	20 (4/20)
Rapid urease test	5 (1/20)
Drug resistance information available from culture, %	30 (6/20)
Amoxicillin resistance (%)	0/6 (0%)
Clarithromycin resistance (%)	2/6 (33.3%)
Metronidazole resistance (%)	0/6 (0%)
Sitafloxacin resistance (%)	3/6 (50%)
Eradication rate, % (95% CI) (FAS), *n*	90.0% (68.3–98.8%), *n* = 20
Eradication rate, % (95% CI) (PPS), *n*	94.4% (72.7–99.9%), *n* = 18

Abbreviations: CI, confidence interval; Diagnosis of *H. pylori* infection, %, diagnosis method for *H. pylori* infection before eradication therapy; Evaluation by *H. pylori* stool antigen test, %, eradication success rate determined by the *H. pylori* stool antigen test; FAS, full analysis set; PPS, per‐protocol set; SE, standard error; UBT, ^13^C‐urea breath test.

### Efficacy

The eradication rates were 90% (95% CI 68.3–98.8, *n* = 20) in the FAS population and 94.4% (95% CI 72.7–99.9%, *n* = 18) in the PPS population. The upper 95% CIs exceeded 80%; we will now proceed to an RCT comparing the VA‐dual and VAC‐regimens.

### Adverse events

The total adverse event rate was 40% (any adverse event in 8 of the 20 cases). All adverse events were mild (CTCAE grade 1). The 11 adverse events were 15% abdominal fullness, 15% nausea, 10% abdominal pain, 5% urticaria, 5% diarrhea, 5% constipation, 5% anorexia, 5% vomiting, and 5% fatigue. All adverse events resolved spontaneously. No patient was hospitalized. The AUQ scores are shown in Table 2. This score is not affected by physician assessments and may reflect the sensitivities of patients to symptoms. All AEQ 3 and AEQ 2 scores were finally assessed as CTCAE grade 1 adverse events by physicians, similar to what we found previously.

**TABLE 2:** Adverse effects of treatment with vonoprazan, and amoxicillin dual therapy assessed by questionnaire

	AEQ 1, 2, or 3 (%)	AEQ 2 or 3 (%)	AEQ 3 (%)
Diarrhea	40	10	5
Dysgeusia	5	5	0
Nausea	15	5	5
Anorexia	15	5	0
Abdominal pain	10	0	0
Heartburn	5	0	0
Hives	10	5	0
Headache	5	5	0
Abdominal fullness	15	10	5
Belching	10	0	0
Vomiting	5	5	5
General malaise	10	0	0
Other	15	0	0

Abbreviations: AEQ, adverse‐effect questionnaire; AEQ1, weak; AEQ2, moderate; AEQ3, strong.

## Discussion

To the best of our knowledge, this is the first interventional study to assess the efficacy and safety of VPZ 20 mg bid and AMPC 500 mg qid 7‐day dual therapy in terms of first‐line *H. pylori* eradication. The eradication rates were good (90–95%) in both the FAS and PPS populations. Only CTCAE grade 1 adverse events were noted; these disappeared after the completion of eradication therapy.

In terms of AMPC dose and frequency, as shown in Table 3, the total AMPC dose and frequency of our regimen (500 mg qid, 2000 mg/day) are higher and more frequent than previous VPZ/AMPC dual therapy regimens. Our regimen and that of Furuta's afforded 90% eradication on ITT analysis^5^; Suzuki's regimen did not attain this figure.^6^ Thus, we think AMPC should be given at 500 mg qid (four times daily) or 500 mg tid (three times daily),^5^ not at 750 mg bid (twice daily).^6^ In terms of the optimal AMPC frequency, PPI‐based dual therapy with AMPC also evidenced higher eradication rate trends in qid than tid. Zhu *et al*. reported a systematic review with meta‐analysis of high‐dose PPI‐amoxicillin dual therapy, and AMPC tid had an unacceptable eradication rate of 73%, whereas AMPC qid showed a more effective eradication rate of 87%.^16^ These PPI‐based dual therapy results indicate that 500 mg qid (this study) has more potential than 500 mg tid (Furuta reported).

**TABLE 3:** Comparison of dual therapy with vonoprazan and amoxicillin for *Helicobacter pylori* eradication

Study	Year	Dosing scheme of amoxicillin	Dosing scheme of vonoprazan	Duration of treatment days	n	Eradication rate (ITT)	Eradication rate (ITT)
						[95%CI]	[95%CI]
This study	2022	500 mg qid (2000 mg/day)	20 mg bid	7	20	90.0% [68.3–98.8]	94.4% [72.2–99.9%]
Furuta	2020	500 mg tid (1500 mg/day)	20 mg bid	7	56	92.9% [82.7–98.0]	94.4% [84.6–98.8]
Suzuki	2020	750 mg bid (1500 mg/day)	20 mg bid	7	168	84.5% [78.2–89.6]	87.1% [81.0–91.8]

We think PPI‐based dual therapy regimens with 500 mg tid or 1000 mg bid AMPC should not replace triple therapy regimens, because, randomized trials have revealed the superiority of triple, as compared to dual therapies.^17^, ^18^, ^19^, ^20^ In addition, many PPI‐based dual therapies did not show sufficient eradication rates.^18^, ^21^, ^22^, ^23^

In terms of the regimen period, we chose 7 days based on Furuta and Suzuki's results. About the reason for 7 days settlement, VPZ enables the intragastric pH to reach around 7 within 3‐4 h and usually, AMPC‐sensitive *H. pylori* cannot survive for 1 week on the agar plates containing AMPC at pH 7.^24^ At the time of designing this study, only 7 days VPZ and AMPC dual therapy were reported. In PPI‐based regimens, a meta‐analysis showed a higher eradication rate with 10 or 14 days compared to 7 days, but a higher adverse events rate with a 1.2 risk ratio (95%CI: 1.06–1.37).^25^ Whether 10 or 14 days is superior to 7 days for VPZ and AMPC dual therapy is still a question. Our hypothesis is that non‐inferiority of 7 days of this study's regimen to the standard 7 days VAC‐regimen in Japan if non‐inferiority is met 7 days dual regimen is more feasible at cost and safety than 10 or 14 days to become insurance covered standard regimen in Japan. We think the comparison between 10 or 14 days and 7 days is the next step research question to improve VPZ and AMPC dual therapy.

In terms of study design, the Furuta work was retrospective^5^ and Suzuki conducted a multicenter randomized trial^6^; our present study was a dual‐center, single‐arm interventional trial designed to show whether an RCT was appropriate. The Furuta work and our work emphasize the potential of VPZ‐based dual therapies for first‐line eradication; these are better than PPI‐based dual therapies. The combination of 500 mg qid AMPC and 20 mg bid VPZ was safe, as was the Furuta 500 mg tid AMPC and 20 mg bid VPZ. The outcomes of PPI‐based 500 mg qid AMPC dual therapy were better than those of PPI‐based 500 mg tid AMPC dual therapy. We are now conducting the next step dual‐center RCT. This study may bring the first RCT result showing non‐inferiority of vonoprazan and AMPC dual therapy to standard VAC‐regimen in ITT analysis.

In terms of antibiotics resistance results, we detected no AMPC resistance in this study (Table 1). The AMPC‐resistance rate is generally very low in Japan.^3^ Thus, VPZ and 500 mg qid AMPC dual therapy can afford a 90% ITT eradication rate against a very low background of AMPC‐resistance. We detected CAM‐ and STFX‐resistance, but no MNZ‐resistance. Generally in Japan, the CAM‐resistance rate is about 33%, and the MNZ‐resistance rate is <5% (as estimated in our review).^3^ Thus, any rescue regimen after dual therapy should contain MNZ.

Regarding the mechanism by which the regimen in this study is effective, we believe that both adequate acid blockade and pharmacologically correct AMPC dosing are key points. VPZ inhibits H+/K+ ATPases rapidly, strongly, and stably compared to PPIs. The intragastric pH increased to over pH 4.0 within 4 h^26^ of VPZ administration and, in 99% of patients, is maintained over pH 5.0.^27^ In addition, the effect is not affected by the CP2C19 genotype.^26^, ^28^ AMPC acts by inhibiting the synthesis of bacterial cell walls and thus, the bacteria must be actively dividing and synthesizing cell walls for AMPC to be effective. At pH > 5.0, *H. pylori* can enter a growth phase, and become more susceptible to AMPC.^29^ Thus, the first mechanism is sufficient acid blockade by VPZ to be AMPC more effective. As for the second mechanism of AMPC dosing, our 4 times daily (qid) dosing enables more %Time > MIC compared to tid or bid, pharmacologically. Effects of AMPC depend on %Time > MIC and not the AUC or Cmax, because antibiotic with a beta‐lactam ring, such as AMPC, exert little post‐antibiotic effects for bacteria including *H. pylori*.^30^

Our study had certain limitations. First, this was a single‐arm interventional study exploring whether to continue to a non‐inferiority RCT comparing 7‐day VPZ 20 mg bid and AMPC 500 mg qid dual therapy with 7‐day triple therapy including VPZ 20 mg bid. Our sample size was small; our planned RCT will feature non‐inferiority testing. Second, AMPC‐resistance information was obtained for only 30% of cases (rate: 0%). However, our recent review showed that AMPC‐resistance is generally very low in Japan^3^; thus, our results can be generalized to settings featuring AMPC‐susceptible *H. pylori*. Our results are meaningful in regions or countries (including Japan) with very low AMPC‐resistance.

In summary, this dual‐center, single‐arm interventional study revealed a good eradication rate, and treatment was safe. We now proceed to an RCT comparing 7‐day VPZ 20 mg bid and AMPC 500 mg qid dual therapy to VPZ‐based triple therapy with AMPC and CAM. A 7‐day VPZ 20 mg bid and AMPC 500 mg qid dual therapy may be better than a 7‐day VPZ 20 mg bid and AMPC 750 mg bid dual therapy.

## Informed consent

All participants provided written informed consent prior to study enrolment.

## Data Availability

Not applicable.
